# ZeBRα a universal, multi-fragment DNA-assembly-system with minimal hands-on time requirement

**DOI:** 10.1038/s41598-019-39768-0

**Published:** 2019-02-27

**Authors:** David Richter, Katharina Bayer, Thomas Toesko, Stefan Schuster

**Affiliations:** Department of Animal Physiology, University of Bayreuth, 95440 Bayreuth, Germany

## Abstract

The recently evolved field of synthetic biology has revolutionized the way we think of biology as an “engineerable” discipline. The newly sprouted branch is constantly in need of simple, cost-effective and automatable DNA-assembly methods. We have developed a reliable DNA-assembly system, ZeBRα (Zero-Background Redα), for cloning multiple DNA-fragments seamlessly with very high efficiency. The hallmarks of ZeBRα are the greatly reduced hands-on time and costs and yet excellent efficiency and flexibility. ZeBRα combines a “zero-background vector” with a highly efficient *in vitro* recombination method. The suicide-gene in the vector acts as placeholder, and is replaced by the fragments-of-interest, ensuring the exclusive survival of the successful recombinants. Thereby the background from uncut or re-ligated vector is absent and screening for recombinant colonies is unnecessary. Multiple fragments-of-interest can be assembled into the empty vector by a recombinogenic *E. coli*-lysate (SLiCE) with a total time requirement of less than 48 h. We have significantly simplified the preparation of the high recombination-competent *E. coli*-lysate compared to the original protocol. ZeBRα is the least labor intensive among comparable state-of-the-art assembly/cloning methods without a trade-off in efficiency.

## Introduction

Seamless multi-fragment DNA assemblies have recently been added to the toolkit of molecular biology. Particularly the Gibson-assembly has demonstrated its exceptional power as shown by the *in vitro*-assembly of the *Mycoplasma genitalium* genome^1^ as well as the mouse mitochondrial genome^2^. “Home-brewed” Gibson-assemblies are widely used but their efficiency rarely comes close to that of the published method. Commercial assembly kits exert superior efficiencies but are costly and thereby not suitable to be used on a large-scale for routine cloning. An affordable highly efficient cloning method with reasonable hands-on time is in high demand by the molecular biological community.

Not surprisingly, a PubMed search for “DNA assembly cloning” resulted in >2300 publications in this field and illustrates the general interest for efficient, fast and robust methods. However, only a few non-commercial methods like LIC^3^, SLIC^4^, SLiCE^5^, Hot-Fusion^6^, Golden-Gate^7,8^ to name a few, reached wider acceptance outside synthetic-biology. This is probably attributable to the specific requirements of different cloning projects, the lack of universality of some of the aforementioned methods, and the effort needed to evaluate and establish novel methods. The ideal method should be versatile, efficient, time and resource-effective and does not require expert skills or expensive reagents and equipment. Some cloning systems like the Golden-Gate^7,8^, Heaven’s Gate^9^, TA-Cloning and TOPO®-Cloning are limited to a single fragment to be cloned at a time. Additionally, most systems critically require gel-purified vectors to reduce the number of bacterial colonies to be screened.

**Figure 1:**
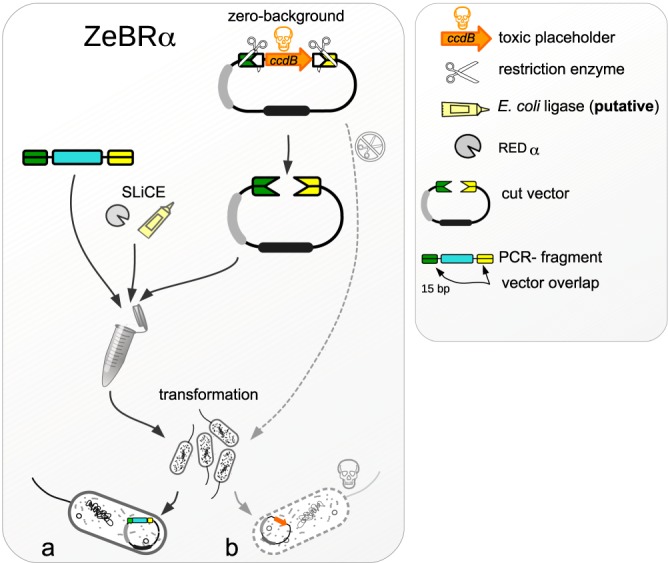
The ZeBRα cloning strategy is the combination of a recombinogenic *E. coli* extract with a “zero-background” vector. (**a**) The strong *in vitro* recombination capacity of the bacterial extract (SLiCE) allows the seamless assembly of DNA fragments. SLiCE uses an *E. coli*-lysate containing Redα-exonuclease and probably endogenous ligase (pictured as grey pie and yellow glue in (a)) to assemble DNA fragments that share about 15 bp of identical sequence at the ends. (**b**) The active selection against undigested plasmid-carryover by a toxic-gene product in the starting vector. Carryover of undigested vector into a ligation and subsequent transformation is the main cause of colonies without insert. The vector used for ZeBRα is carrying a *ccdB*-toxin coding sequence that is replaced by the fragment(s) to be cloned. Undigested or re-ligated vector will kill the transformed cloning strains of *E. coli*.

In search of a method that unites the advantages of previous methods for robust and high-throughput cloning, we have developed a novel strategy that combines a multi-fragment seamless assembly method with positive selection for the desired cloning products. Our method, named ZeBRα (Zero-Background Redα), can be used for assembling multiple fragments without the need to screen for the desired recombinant clones. The method can be adapted to any cloning task and can be used in high-throughput approaches. We exploit the recently developed, highly recombinogenic cell-extracts of *E. coli* (PPY-strain^5^, NEB 5-alpha) to overcome the limitation of cloning a single DNA-fragment at a time (Fig. 1a). First, we simplified the preparation of the extract considerably, compared to the original protocol^5^ by using an arabinose-autoinduction medium. The recombinogenic components are released from the PPY-cells upon lysis with mild detergents. We have optimized the lysis, Redα-induction, and composition of the assembly-reaction and identified critical steps for greater reproducibility.

Secondly, we harnessed a vector with the *ccdB*-suicide cassette, facilitating the recovery of the desired recombinant clones by counter-selecting against uncut or re-ligated vectors (Fig. 1b). The toxic-placeholder is replaced by the DNA-fragments to be cloned, ensuring that only the transformants with recombinant plasmids survive after transformation. The hands-on time for cloning is thus greatly reduced by disposing of two time-consuming steps – gel purification of the vector and screening for colonies. This benefit becomes apparent by a simple calculation. Clone verification, the final step of cloning, is one of the most laborious steps in the procedure. It is common practice to analyze between 4 and 20 clones for a single recombinant vector. So for a typical number of 10 to 100 cloning experiments the laborious final step needs to be carried out up to 2000 times, making it plain that time and resources can significantly be saved by any improvement in the verification step.

In the final embodiment of the method we demonstrate that with the optimized conditions an extract from the common DH5α-strain derivative NEB 5-alpha can fully replace the PPY-cells, thereby further simplifying the overall process.

## Results

### Optimization-strategy of recombinogenic PPY-extract preparation

**Figure 2:**
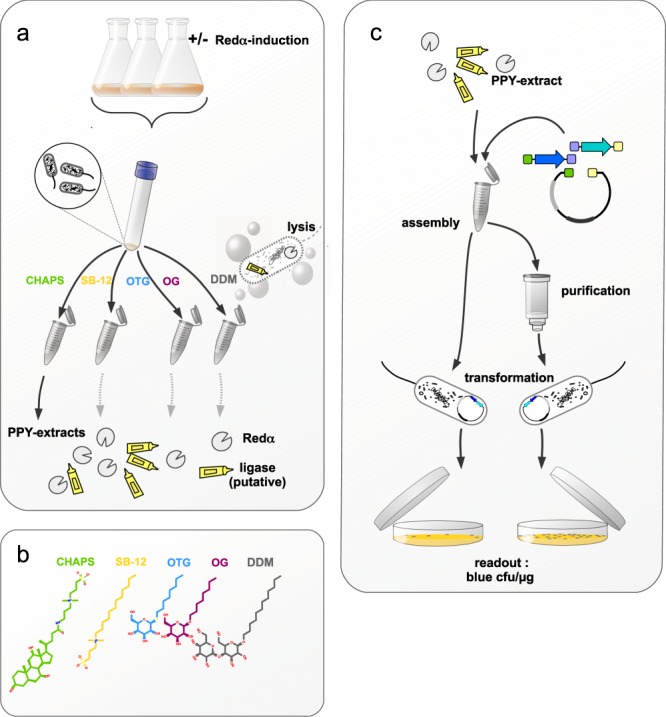
Strategy for SLiCE optimization and evaluation. (**a**) Flow chart of the optimization process for generating a recombinogenic *E. coli* lysate. The PPY strain is a DH10B-derivative used to prepare the recombinogenic cell lysate and expresses the coding sequences for Redαβγ. The extracts, derived from arabinose autoinduced PPY-cells, were compared to extracts made from non-induced PPY-cells. (**b**) Structure of the examined non-ionic detergents used to prepare the recombinogenic PPY-extracts, CHAPS, Sulfo-Betain (SB-12), n-Octyl-β-D-thioglucopyranosid (OTG), n-Octyl-β-D-glucopyranosid (OG) Dodecyl-β-D-maltosid (DDM). (**c**) PPY-extracts were tested for their recombination capacity by assembling three DNA fragments with overlapping ends, to generate a recombinant plasmid constitutively expressing a blue chromoprotein. To examine the effects detergents had on the transformation, samples were split after the assembly reaction. One part was transformed into NEB 5-alpha unpurified; the other fraction was purified by silica-column chromatography prior to transformation.

Experiments were designed carefully to evaluate the contribution of different factors to the recombination success of the SLiCE-assembly. With the intention to find the simplest conditions for the preparation of the highly-recombinogenic PPY-extract, we decided to examine the necessity of Redα-exonuclease expression for the recombination capacity of the PPY-cell extract. First, cell lysates prepared from PPY-cells where Redα was induced were compared to extracts where Redα was not induced (Fig. 2a). The evaluation of Redα-induction was chosen because Redα-exonuclease is seen as the key component in the recombination process^5^, but *E. coli* strains with multiple exonuclease deletions were shown to outperform their wild-type counterparts in recombineering experiments^10^. Additionally, several studies, including the original SLiCE-publication^5^ used bacterial-extracts from DH10B or JM109 *E. coli* for *in vitro* DNA-assemblies without Redα-exonuclease successfully, yet significantly less efficient^5,11–15^. The second point we addressed was how the performance of the cell-extracts was affected, depending on the type of detergents used. We chose a small variety of five nonionic and zwitterionic detergents respectively (Fig. 2a–c). Nonionic and zwitterionic detergents are widely used for bacterial cell lysis in protein purification^16^, because they disrupt the bacterial cell wall with minimal adverse effects on protein structure and function. Finally, since chemically competent *E. coli* cells are very sensitive to detergents^17^, we tested if removing the detergent from the DNA-assembly reaction prior to transformation improves the overall number of recombinant colonies. To quantify the recombination capacity of the different PPY-extracts, we adopted the method originally developed by Fisher and colleagues^12^: The assembled PCR-fragments result in a vector constitutively expressing a blue chromoprotein when grown on a selective medium. The promoter and the coding-sequence for the blue chromoprotein were contributed by separate PCR-fragments, thereby ensuring that only successful recombinants resulted in blue colonies on kanamycin-plates (Fig. 2c).

The resulting optimized preparation protocol of the recombinogenic PPY-extract was combined with a “zero-background” vector we created, to establish the ZeBRα- (Zero-Background Redα) assembly-method.

### Lambda Redα-autoinduction increases the recombination capacity of PPY-extracts

The PPY-strain used for preparation of the recombinogenic extracts has a genomically integrated Red-system of bacteriophage λ. It mediates induced homologous recombination in *E. coli* and consists of Redα-exonuclease, Bet, and Gam proteins,  it is widely utilized technically for the recombineering-technology (for review see^18,19^). While the expression of Bet and Gam are under control of constitutive promoters, the Redα-exonuclease is arabinose inducible^5^. Due to its high recombinogenic capacity the PPY-extract offers a real alternative to the commercial assembly-systems. The drawback of the SLiCE-method is, that the preparation of a PPY-extract requires significant hands-on time and skill to induce the Redα-exonuclease. Therefore, our first goal was to simplify the preparation of the cell extract. PPY has been described to grow slowly in 2xYT-broth^5^, making the manual induction of Redα-exonuclease expression lengthy. We hypothesized that arabinose autoinduction medium^20^ might offer a more user-friendly method of preparing the cell extract. Autoinduction is self-limiting, without the constant need of monitoring the cell density, thereby massively reducing hands-on time and error rate. We compared the recombinogenic capacity of extracts derived from PPY-cells grown in arabinose autoinduction medium to cells grown in a medium where arabinose has been replaced by lactose (non-inducing). We pooled six independently fermented autoinduced PPY-cultures into one sample and four independently fermented non-induced PPY-cultures into the control sample.

The pooled induced- and control-samples were pelleted and equal aliquots of the pellets were extracted with one of the following nonionic sugar-glucosides (n-Octyl-β-D-thioglucopyranoside OTG, n-Octyl-β-D-glucopyranoside OG, Dodecyl-maltoside DDM) or zwitterionic (Sulfobetaine SB12, CHAPS) detergents. By pooling independently fermented PPY-cultures the differences between the autoinduction and control are made dependent mainly on the process of the autoinduction itself but not on handling or downstream processes. The resulting reduction in variation comes at the cost of more care being required in interpreting statistical values because independent samples are combined to just one.

**Figure 3:**
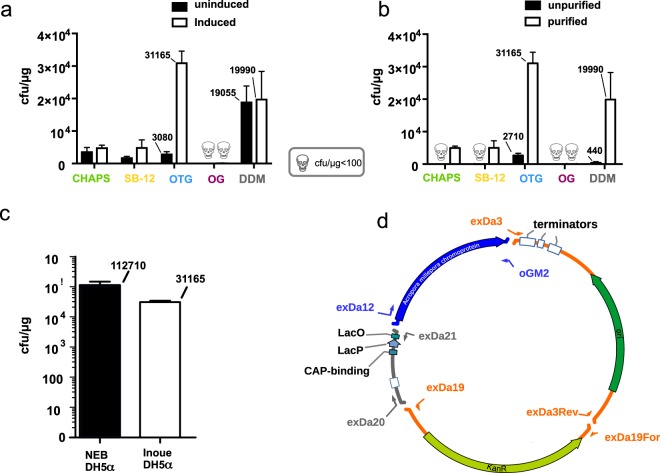
Comparison of the influence of detergent, autoinduction, post-assembly purification and competency of used bacteria on DNA assembling efficiency. (**a**) In four of the five PPY lysis conditions induction of Redα had moderate effects. Five different detergents were tested on PPY-cells grown with either lactose (Redα un-induced) or arabinose (Redα induced). All assemblies were column-purified before transformation into NEB 5-alpha. Bars indicate standard error of three independent replicates of an assembly reaction in all following graphs. PPY recombinogenic capacity was assessed in three-fragment assemblies. (**b**) Column-purification of the three-fragment-assembly reactions led to markedly increased number of recombinant colonies for all tested detergents. In the case of CHAPS and SB-12, unpurified samples resulted in no colonies. The OTG derived PPY-extract resulted in the highest number of recombinant colonies without purification. All assemblies were arabinose-induced. (**c**) Chemical competency has profound influence on recombination efficiency. OTG prepared PPY-lysate was used in a three-fragment-assembly and transformed into commercial NEB 5-alpha competent *E. coli* (1 × 10^9^ cfu/µg pUC DNA) or the same strain prepared by the Inoue-method^21^ (2.3 × 10^6^ cfu/µg DNA). (**d**) For convenient readout of the potency of the PPY-extracts PCR-fragments used for the three- and four-way assembly reactions consisted of a blue chromoprotein coding ORF, a kanamycin resistance gene an origin-of-replication (on one fragment for the three-fragment assembly) and a bacterial basal-promoter-fragment. Only successful recombinants could produce blue colonies on kanamycin plates. The PCR-fragments to be assembled had overlapping bases that summed up to about 15 bp overlapping ends.

PPY-cell extracts that were derived from the arabinose induced PPY-cells resulted in more recombinant colonies for the three-fragment assembly than extracts prepared from non-induced PPY-cells across all tested detergents (Fig. 3a). Induced PPY-cells extracted with OTG produced the extract with the highest total recombinogenic capacity among all tested detergents (Fig. 3a) with 3 × 10^4^ cfu/µg transformed DNA. The differences in recombination capacity are likely the result of an interaction between autoinduction and the ability of the detergents to solubilize PPY-components required for effective recombination. The extracts prepared with OTG and DDM outperformed the CHAPS and SB-12 extracts by a factor of 6 and 4 respectively, yielding roughly 3 × 10^4^ and 1.9 × 10^4^ cfu/µg. OG derived PPY-extract yielded only 85 colonies. There are several possible interpretations, including that the detergent could not be removed by column purification, that OG does not effectively solubilize components from the PPY-cells, relevant for recombination or that OG extracted components that interfere with successful recombination. Remarkably, DDM extraction of induced and autoinduced PPY resulted in almost identical numbers of recombinants and ranked second highest among all tested detergents. Therefore, DDM seems to extract components promoting recombination independently of Redα-exonuclease more effectively than the other detergents or releases fewer proteases or nucleases interfering with SLiCE. To test the effect of autoinduction on the recombination capacity of PPY-extracts the detergents were removed from the test-assemblies before transformation. In order to examine if this extra step is crucial, in the following we tested the effect of the detergents on the consecutive transformation by omitting the purification.

### Removal of the detergent from the assembly reaction is essential prior to transformation

Half of the recombination-reactions with PPY-lysates from autoinduced cells were subjected to silica-column purification prior transformation. The rest of the reaction mixtures were transformed without prior purification. Unpurified assembly-reactions resulted in a dramatic reduction of colonies with assembled plasmids. OTG and DDM resulted in an average of 2700 and 440 recombinant colonies per µg transformed DNA corresponding to a ten-fold and 43-fold reduction of recombinant colonies, respectively (Fig. 3b). For unpurified CHAPS and SB-12 containing assembly-mixes, the colony count was reduced from roughly 5000 recombinants/µg after column-purification to less than 50 without purification. This demonstrates that there was sufficient detergent present in the transformation reaction to lyse the competent cells or interfere with the transformation. With the exception of OG, which seemed to be resistant to removal by silica-columns, all other detergents were removed by the column-purification procedure. The OTG prepared PPY-extracts exerted the best overall performance resulting in about 2700 recombinants/µg transformed DNA without purification. This indicates a low toxicity or interference with the recombination and transformation processes. The OTG presumably releases some of the cellular protein content of the PPY-cells without entirely disrupting them. An additional benefit of the column purification might be the removal of proteins from the assembly-mixture that potentially inhibit transformation. For all subsequent experiments, we used assemblies from autoinduced PPY-cells extracted with OTG and subjected them to column purification prior transformation.

### The competence of the *E. coli*-cells transformed with the assembly-reaction critically affects the number of recombinants

The quality of competent cells is a limiting factor for efficient DNA-cloning. Commercial, highly competent *E. coli* cells (1 × 10^9^ cfu/µg) are ideal for multi-fragment DNA–assemblies, but are the most expensive reagent used in the reaction.

Since different authors used commercially available highly competent cells (2 × 10^8^ cfu/μg pUC19 DNA^13^), or electrocompetent DH10B^5^, we investigated if the recombinogenic PPY-extract would efficiently assemble three-fragments with “home-brewed” chemically competent NEB 5-alpha prepared by the Inoue-method^21^. Our preliminary experiments indicated, that the NEB 5-alpha strain was a superior recipient for the assemblies compared to chemically competent DH10B (data not shown). Chemically competent NEB 5-alpha prepared by the Inoue-method in our laboratory usually reach competencies of about 3 × 10^7^ cfu/µg. The competency of the NEB 5-alpha batch used throughout experiments of this study was 2.3 × 10^6^ cfu/µg. We decided to use cells from a batch with sub-optimal competence, to test the efficiency of the SLiCE extract under more stringent conditions. As in the previous experiments, we assembled three PCR-fragments and transformed half of the column-purified reaction into commercially available NEB 5-alpha (10^9^ cfu/µg) and NEB 5-alpha, made competent by the Inoue-method (2.3 × 10^6^ cfu/µg). The competence of the commercial NEB 5-alpha cells was about three hundred times greater than the competence of the cells made competent by the Inoue-method and translated into about 35-times more recombinant colonies (Fig. 3c). The competence of the cells transformed with the assembly reactions had a significant impact on overall recombination efficiency.

### Assembling four PCR-fragments markedly reduces the recombination success compared to three fragments

**Figure 4:**
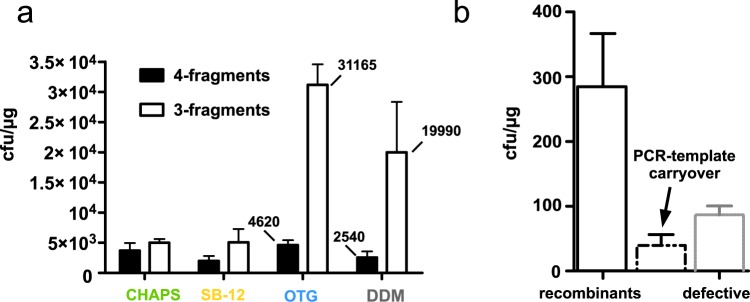
Three or four fragments can efficiently be assembled with PPY-cell extracts, while iVEC/“transformation-cloning” with three fragments is markedly less efficient. (**a**) The assembly of four fragments in a single reaction reduces the number of successful assemblies by a factor of two to ten as compared to the three-fragment-assembly. Cell-extracts were prepared from autoinduced PPY cells and the assemblies were purified prior transformation. (**b**) Assembly of three fragments by iVEC/“transformation-cloning” with NEB 5-alpha resulted in roughly 250 recombinant colonies/µg transformed DNA. A significant number of colonies harboring plasmids with defective inserts (grey bar) and PCR-template carry-over (dotted bar) were present on the plates.

Next, we tested the performance of the cell extracts derived from autoinduced PPY extracted with OTG, for their ability to perform an *in vitro* assembly of four PCR-fragments. The original SLiCE-method^5^ demonstrated an impressive number of DNA-fragments joined in a single reaction. We examined, how the number of fragments being assembled in a single reaction affects the overall number of recombinant colonies. Multiple factors influence the efficiency of *in vitro* assemblies as the size, fragment-number, sequence, GC-content of the DNA-fragments, the insert-to-vector ratio as well as the length of overlapping bases (see^22–30^). The overall size of the assembled plasmid likely adds up to this. We have designed an additional set of primers that generates two DNA-fragments from the vector-fragment of the three-fragment assembly (Figs 3d and S1). Thereby the four-fragment assembly and the three-fragment assembly differ only in the number of fragments. We have tested the efficiency of the PPY-preparations for the assembly of three and four fragments respectively since this is a range that is both useful and sufficient for most cloning projects. We noticed a marked drop in recombinant colony numbers when four fragments were to be joined as compared to the three-fragment assembly (Fig. 4a). The reduction in the number of recombinant colonies was about six-fold for OTG. Interestingly, CHAPS derived extract of PPY-cells ranked among the least in the three-fragment assemblies but was second best in the four-fragment assemblies, resulting in 73% of the recombinants colonies compared to the corresponding three-fragment assemblies. The OTG derived PPY-extract still outperformed all other detergent extracts in absolute recombinant colony count. The number of recombinant colonies was well over what we would consider being required for standard cloning applications for all tested conditions. This demonstrates the extraordinary recombination capacity and robustness of PPY-extracts.

### PCR-products can be efficiently assembled *in vivo* by “home-brewed” chemically competent NEB 5-alpha –cells

Our goal was to compile a method that minimizes the labor-intensive steps to a minimum. The simplified method to prepare a highly recombinogenic PPY-extract by autoinduction dramatically reduces the need of human assistance during fermentation of PPY-cells. We were wondering if using iVEC/iVEC/“transformation-cloning” would be an even simpler approach for a seamless multi-fragment assembly to be combined with a zero-background vector. Therefore we first assessed how efficiently three fragments could be assembled by the technically simple iVEC/“transformation-cloning” into Inoue-competent NEB 5-alpha (Fig. 4b) By iVEC/“transformation-cloning” we are referring to a set of closely related methods that rely on the endogenous capacity of *recA* cloning strains, such as DH5α or DH10B, to assemble DNA fragments with about 15 bp overlap *in vivo* (SEFC^23^, Co-transformation-cloning^31^, IVA^32^, AQUA^25^, Optimal-Cloning^26^, FastCloning^28^, IVC^30,27,33,34^). We incubated column-purified linear vector and the PCR-fragments at room temperature for thirty minutes followed by iVEC/iVEC/“transformation-cloning” into chemically competent NEB 5-alpha cells.

For a three-fragment assembly we found an average of more than 250 recombinant colonies per microgram transformed DNA, but to our surprise a significant number (about 80) white colonies appeared on the plates. Our strategy does only allow blue recombinant colonies to grow on the plates, so we sequenced a few white colonies and found defective inserts in them (data not shown). We have also noticed a smaller fraction of red colonies corresponding to PCR-template carryover that was not detectable in the PPY-extract mediated assemblies (SLiCE). We conclude that the iVEC/“transformation-cloning” with three-fragments is a valuable method but the colonies need to be screened for correct inserts. Although iVEC/“transformation-cloning” wins over by its simplicity compared to SLiCE it has markedly reduced recombination capacity.

### Plasmid assembly with the ZeBRα-vector pT7-HindIII-ccdB is versatile, simple and effective

**Figure 5:**
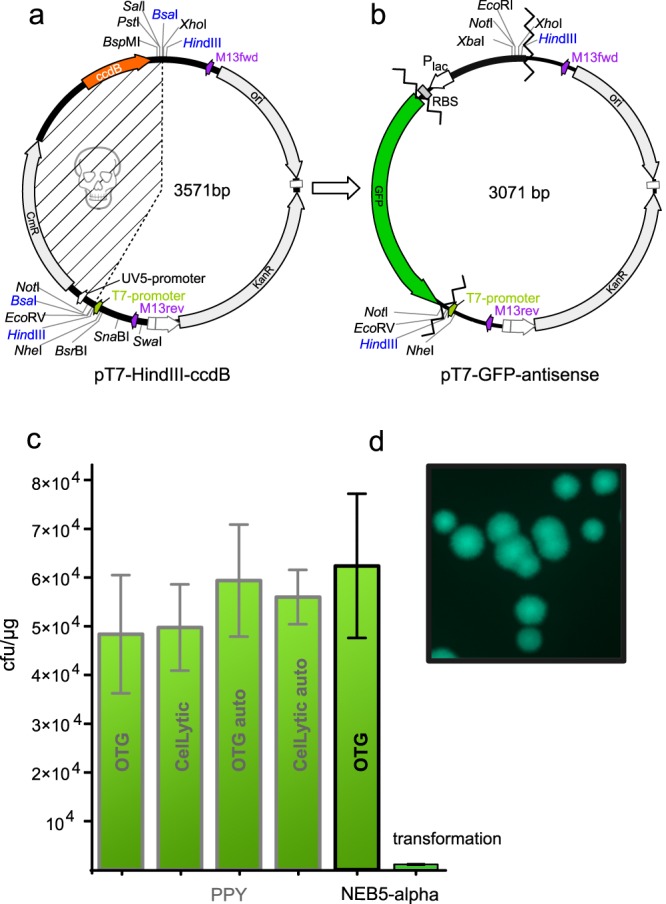
The recombinogenic capacity of OTG extracts from autoinduced PPY and NEB 5-alpha extracts are equivalent or better than PPY-extracts generated by the original protocol. (**a**) Plasmid map of pT7-*Hin*dIII-*ccdB* used to assess three-fragment ZeBRα assemblies. Two *Hin*dIII and two *Bsa*I sites flank the toxic-placeholder-*ccdB*, allowing linearization and removal of *ccdB*. Unique sites are available on either side of *ccdB*. Chloramphenicol-acetyl-transferase coding gene (*CmR*), is part of the placeholder cassette and prevents *ccdB*-loss during plasmid propagation. The hatched region encompasses the fragment removed during cloning. (**b**) Map of the vector pT7-GFP antisense resulting from the three-fragment test-assembly of the pT7-*Hin*dIII-*ccdB* as recipient for a GFP-ORF and a bacterial promoter containing PCR-fragment, to evaluate the efficacy of the ZeBRα-procedure. Criss-cross lines mark the fusion-sites of the assembled fragments. (**c**) Comparison of the recombination capacity of extracts prepared with OTG or CelLyticB^TM^ from manually induced and autoinduced (denoted as “auto” in the column) PPY-cells and NEB 5-alpha. The iVEC/“transformation-cloning” of the respective fragments is shown as last column. (**d**) Green fluorescent NEB 5-alpha colonies harboring the constitutively GFP-expressing vector pT7-GFP antisense.

After optimizing the preparation of a highly recombinogenic PPY-extract, we utilized it to create the ZeBRα-assembly method. We measured how efficiently we can assemble one (Fig. S2) or two PCR-fragments (Fig. 5a,b) into the newly created “zero-background” vector pT7-*Hin*dIII-*ccdB*. In the pT7-*Hin*dIII-*ccdB* two *Hin*dIII and *Bsa*I sites flank the *ccdB*-gene, allowing convenient linearization and removal of the toxic-placeholder *ccdB* (Fig. 5a). Cloning strains (e.g. DH5α, DH10B, JM109, XL-1blue) are sensitive to the *ccdB* gene product, which prevents the survival of cells that were transformed with “uncut” or re-ligated plasmid vectors. Uncut and single-cut-re-ligated vector bearing cells make up for the majority of colonies found on plates after ligation and transformation. Therefore, using a “zero–background” vector eliminates or drastically reduces the need to screen for recombinant clones. Our main goal was to reduce the need of human intervention in creating highly recombinogenic *E. coli* extracts. Therefore we have identified the most critical factors and optimized the induction and extraction process of PPY-cells. We have also demonstrated that the SLiCE-extracts can be used with a positive selection vector, thus significantly reducing hands-on time for multifragment seamless cloning. Finally we wanted to bring everything together and test if our modified method would stand a direct comparison with the original SLiCE method.

For comparing our SLiCE-derivative to the original method we have used pools of independently fermented PPY-cells and induced λ-exonuclease by timed manual addition of arabinose or by using arabinose autoinduction medium. The resulting cells were subjected to detergent extraction with the commercial CelLyticB^TM^-reagent or with OTG. In contrast to the original method we used chemical transformation and a three-fragment assembly into pT7-*Hin*dIII-*ccdB* to make SLiCE extract performance comparable to the ZeBRα-method.

The *Bsa*I linearized pT7-*Hin*dIII-*ccdB* (Fig. 5a) vector was assembled with a 760 bp PCR-fragment encoding the mammalian codon optimized GFP-ORF and a 280 bp fragment providing a Lac-promoter and a bacterial ribosome binding site. The assembled pT7-GFP-antisense was assembled with PPY-extracts or NEB 5-alpha extracts as described above and all assemblies were purified on silica-columns prior transformation. For the iVEC/“transformation-cloning”-method, the column-purified linear vector and the PCR-fragments were incubated at room temperature for thirty minutes and transformed into chemically competent NEB 5-alpha cells. The resulting colonies with successfully assembled plasmids were identified by their intense green fluorescence.

We counted 59371 cfu/µg (N = 8) for the OTG-extract derived from autoinduced PPY-cells and 55985 cfu/µg (N = 3) for the CelLyticB^TM^ extract derived from autoinduced PPY-cells, indicating that OTG and CelLyticB^TM^ have comparable capacity to extract relevant enzymes from the PPY-cells (Fig. 5c). This was also seen in extracts from PPY-cells where the λ-exonuclease was induced by timed manual addition of arabinose (OTG 48361 cfu/µg (N = 4), CelLyticB^TM^ 49735 cfu/µg (N = 4)) (Fig. 5b). Overall the manually induced extracts produced about 5000–10000 less cfu/µg recombinants than the corresponding autoinduced extracts. To our surprise the OTG-extract derived from NEB 5-alpha resulted in the highest number of recombinants 62389 cfu/µg (N = 3) (Fig. 5c). Finally, the iVEC/“transformation-cloning”-method resulted in 1061 cfu/µg (N = 3) transformants, but as previously observed for this method (Fig. 4b) the percentage of GFP expressing colonies was with 30% of the total number of colonies on the plate markedly lower than for the plasmids assembled by bacterial extracts *in vitro*, reaching 99–100% GFP-expressing colonies. Thereby the *in vitro* assemblies resulted in roughly 37 times more colonies compared to iVEC/“transformation-cloning”. For all experiments shown in Fig. 5 we used one batch of “home-brewed” competent NEB 5-alpha prepared by the Inoue-method with a competence of 2.5 × 10^8^ cfu/µg transformed pT7-GFP-antisense. This explains the slightly higher assembly rates compared to the previous three-fragment assemblies shown in Fig. 4. Our OTG-extracts from NEB 5-alpha cells performed equivalently well or even slightly better with pT7-*Hin*dIII-*ccdB* than PPY-extracts.

The exceptionally good performance of NEB5-alpha OTG extracts for the three-fragment assembly lead us to investigate if the recombinogenic capacity of the extracts could be further increased by using other detergents. We have additionally included JM109 cell extracts that were shown to have an extraordinary inherent recombination capacity^5,35^. For this purpose we have used the same panel of detergents as for the PPY cells. NEB 5-alpha cell-extracts showed decent recombination capacity over all tested detergents examined (Fig. S3). For this NEB 5-alpha OTG-extract preparation- assembly combination we measured an average of blue colonies 1.69 × 10^4^ cfu/µg corresponding to 98% of the colonies on the plate. Remarkably the number of recombinant colonies for most detergents was higher for the NEB 5- extracts than the corresponding PPY extracts (Figs 5 and S3). CelLyticB^TM^ derived extracts showed modest fidelity and overall performance with 1.3 × 10^4^ cfu/µg blue colonies representing 83% of all counted colonies. In contrast to the PPY-extracts where SB-12 derived extracts did not show any noteworthy activity, the SB-12 extracts from NEB5-alpha yielded 3 × 10^4^ cfu/µg blue  colonies equivalent to 89% total colonies.

In case of the JM109 CelLyticB^TM^ (2.2 × 10^4^ cfu/µg) and OTG (2.8 × 10^4^ cfu/µg) derived extracts produced the largest numbers of blue recombinants representing 88% and 96% of the overall colonies. We have noticed an increased variance between the technical replicas as can be seen in Fig. S3, that we attribute to variability of the column-purification that we have noticed in some cases.

Our data confirm, that highly recombination competent SliCE-type extracts can be made from different *E. coli* K12 strains and that OTG is an overall good choice due to the high fidelity and high number of recombinants thereby representing the simplest embodiment of the method.

### The toxic placeholder is essential if using unpurified digested vector for multiple fragment assemblies

**Figure 6:**
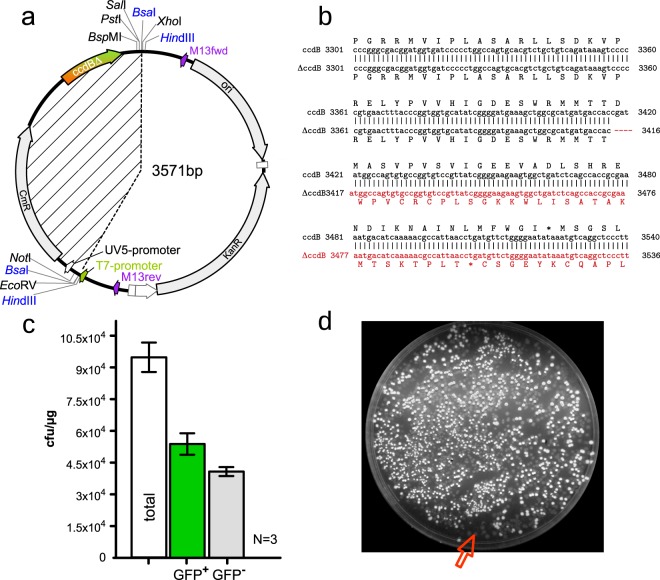
Rendering the CcdB in the placeholder non-toxic increases the number of GFP^–^ background colonies markedly, showing that ccdB is an essential element if working with non-gel purified vector. (**a**) The pT7-*Hin*dIII-dead-*ccdB* differs by a four base-pair deletion in the *ccdB*-coding sequence from it’s predecessor pT7-*Hin*dIII-*ccdB*. The dotted lines encompass the region removed during cloning. (**b**) Sequence alignment of the region encompassing the small deletion in the *ccdB*-ORF, in pT7-*Hin*dIII-dead-*ccdB* compared to the region in pT7-*Hin*dIII-*ccdB* and the resulting frameshift rendering the ΔCcdB non-toxic for *E. coli* NEB 5-alpha. The numbers denote bases in the vector. (**c**) The three-fragment assembly as shown in Fig. 6c uses the pT7-HindIII-dead-*ccdB* vector and OTG-derived PPY-extract to assemble pT7-GFP antisense analogous to the assemblies shown in Fig. 5. The percentage of GFP^+^ colonies drops from nearly 100% for pT7-*Hin*dIII-*ccdB* to 57% for pT7-*Hin*dIII-dead-*ccdB*. (**d**) Image of the mixture of GFP^+^ and GFP^−^ resulting from the assembly (100 µl outgrowth medium spread). The red arrow points at a cluster of GFP^−^ colonies representing un-digested vector that would have to be screened for in a non-model assembly.

We sought to further decompose the ZeBRα-method to keep only the essential elements for maximum convenience, by testing if the *ccdB*-gene in the placeholder cassette could be omitted. Due to their orientation the *Bsa*I recognition-sites are incompatible, and the empty vector does not re-ligate. Thereby, theoretically the *ccdB* would not be essential for a near zero-background assembly. Additionally a *ccdB*–free vector could be propagated in regular *E. coli* strains. We have created a *ccdB*-version that is rendered non-toxic by a small deletion, resulting in a frame-shift and a premature stop codon (Fig. 6b). It has been demonstrated that the amino acids 99–101 in the C-terminus of CcdB are critical for the toxicity of the protein^36^. We used the *Bsa*I-digested, column-purified pT7-*Hin*dIII-dead-*ccdB* as recipient for the promoter fragment and the GFP-ORF in an analogous assembly as shown in Fig. 5. The assemblies resulted in over 9 × 10^4^ colonies per µg transformed DNA in total, when pT7-*Hin*dIII-dead-*ccdB* was used. As expected over 40% of colonies were GFP^−^. We assume that this is the result of incomplete digestion of the vector that occurs in virtually every digestion. We conclude that the toxic-placeholder cassette is essential for convenient assemblies and should not be omitted for the sake of less colony screening or the vector should be gel-purified.

### Chemically competent autoinduced PPY-cells are not suitable for assembling multiple DNA fragments *in vivo*

The high success rate of *E. coli* extract-mediated assemblies from PPY-cell extracts and NEB 5-alpha “transformation-cloning” provoked the question if the convenience of “transformation-cloning” could be combined with the accuracy and efficiency of the SLiCE-method. λ-Red recombineering with chemically competent cells is known to be inefficient compared to the use of electroporation as described earlier^37^. The reason for the reduced success could be due to the generally reduced health of the cells harboring λ-Red recombination machinery, or the interference of λ-Red induction and the induction of chemical competence.

**Figure 7:**
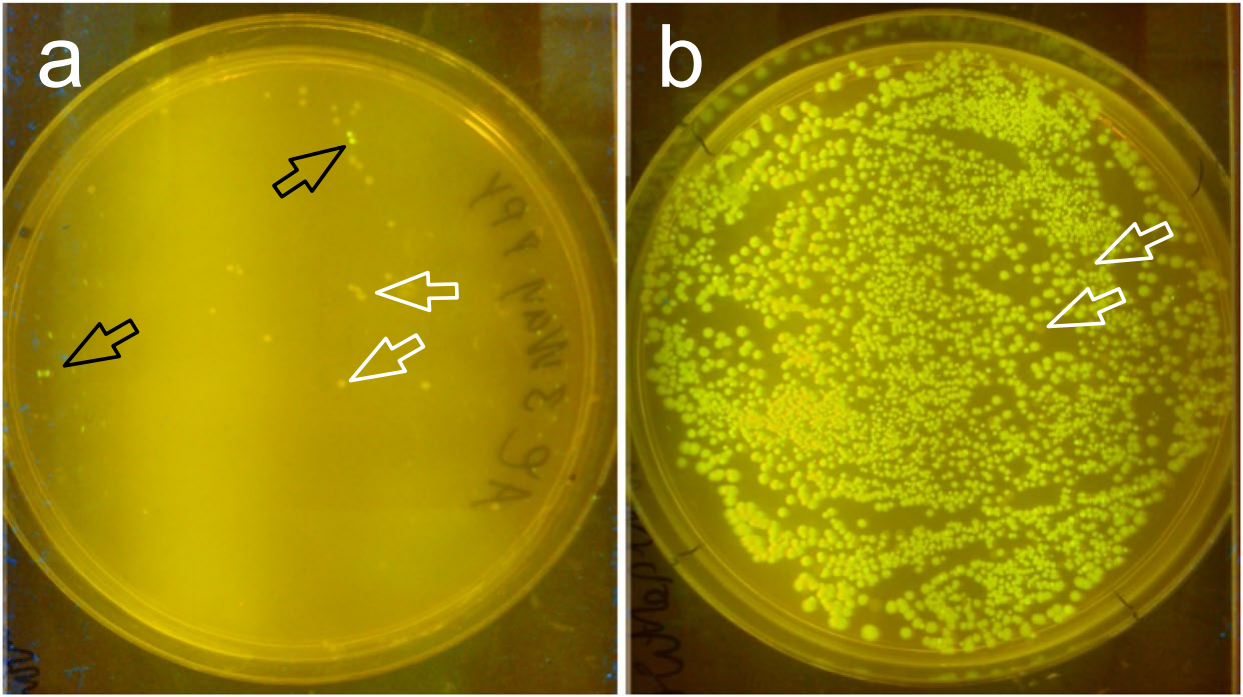
Arabinose induced chemically competent PPY-cells are not suitable for iVEC/“transformation-cloning”. (**a**) An example plate is shown with green fluorescent colonies resulting from iVEC/“transformation-cloning” with chemically competent, arabinose-induced PPY. We used pT7-*Hin*dIII-*ccdB* in the three-fragment assemblies as shown in Fig. 5, resulting in the GFP-expressing vector pT7-GFP antisense as readout. (**b**) Green fluorescent colonies resulting from ZeBRα-cloning using OTG extracts of autoinduced PPY. White arrows point at non-fluorescent colonies, black arrows in (a) point at GFP^+^ -colonies.

We started by comparing two well-established methods to induce chemical competency of PPY-cells (“Inoue-method” and “Hanahan-RbCl-method”) but we were not able to reach a competency of 10^6^ cfu/µg transformed supercoiled DNA with either method (data not shown). Finally with CCMB80-Buffer we were able to get arabinose-induced λ-exonuclease PPY-cells with a competency of 8 × 10^7^ cfu/µg. The growth phase in which cells are exposed to the buffers inducing chemical competency is critical. In our hands competency peaked at lower OD_600_, between 0.2–0.4. According to the original protocol arabinose induction of the λ-Red in PPY-cells is supposed to be done at OD_600_ = 0.5 for 2 h. This would mean that the cells would have passed their window of maximal competence. We therefore induced the PPY cultures in 2xYT (supplemented with glycerol) at OD_600_ = 0.13 and incubated the cells till the OD_600_ reached 0.37, before we started the protocol to induce chemical competency. Since there are recombineering protocols including arabinose in the culture medium at the time point of inoculation we assumed this strategy might be a compromise. Unfortunately PPY cells did not provide a noteworthy number of recombinant colonies when we tested them with ZeBRα-vector in a three-fragment assembly. Additionally most of the colonies were defective as can be seen in Fig. 7a white arrows and Tables S1 and S2.

PPY did not exhibit any significant recombination capacity *in vivo* compared to NEB 5-alpha, resulting in few defective colonies, which makes PPY impractical to use for this purpose. Additionally the overall procedure is significantly more laborious and prone to error by the operator, compared to all previously described methods. Our result fits to the previous observation that λ-Red mediated iVEC/“transformation-cloning” critically depends upon electroporation^38^. Further attempts to improve the outcome of λ-Red mediated iVEC/“transformation-cloning” with chemically competent un-induced PPY-cells had a similar outcome. We used CCMB80 competent un-induced PPY-cells and supplemented the outgrowth-medium with arabinose after the heat shock-step of transformation, with slightly better results as for the autoinduced chemically competent cells (data not shown).

### High-fidelity five fragment seamless ZeBRα-assembly

**Figure 8:**
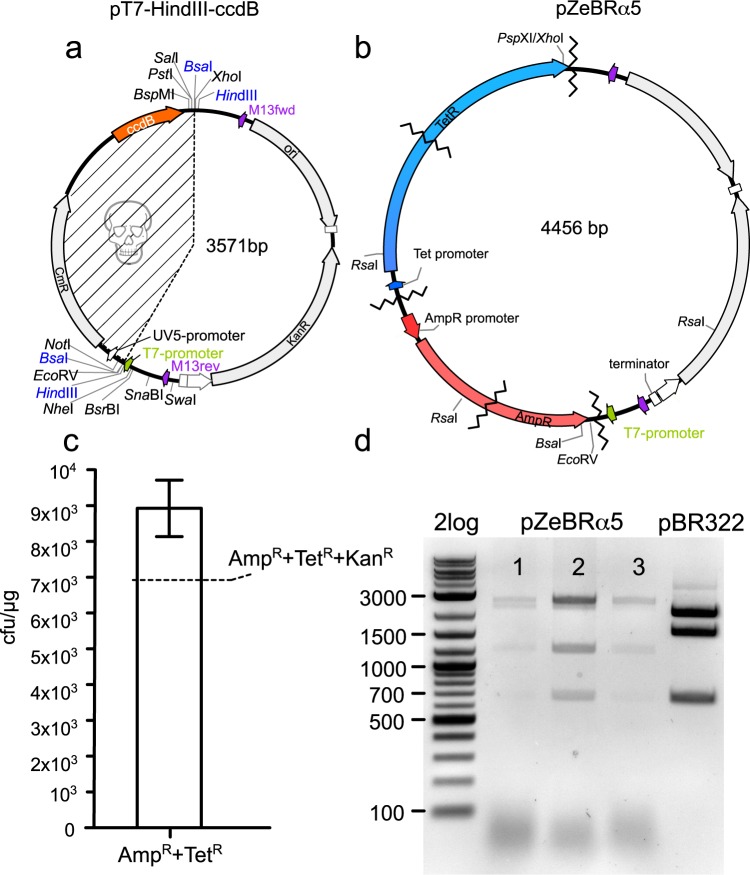
Effective, high fidelity five-fragment ZeBRα-assembly with OTG derived autoinduced PPY-extracts. (**a**) ZeBRα assembly of four fragments into pT7-*Hin*dIII-*ccdB* results in the triple-antibiotic resistant pZeBRα5 for convenient screening of successful recombinants. (**b**) The resistance against ampicillin and tetracycline critically depends on in-frame fusion of the respective ORF and serves therefore as estimate for the error-rates of the recombinogenic extracts. (**c**) Colonies displaying the double and triple antibiotic phenotypes expressed as cfu/µg transformed DNA expected for PCR-template carryover (pBR322) and the pZeBRa5 (dashed line). (**d**) Gel image of *Rsa*I digested plasmids prepared from triple resistant colonies and the pBR322-vector that was used to generate the PCR-fragments for the assemblies.

The high efficiency by which OTG derived bacterial extracts were able to join two-fragments with pT7-*Hin*dIII-*ccdB* made it plausible that the upper limit of fragment number was not yet reached. We developed a test-assembly that would additionally lay more emphasis on the fidelity of the reaction and would allow testing how efficient four fragments could be seamlessly joined into our pT7-*Hin*dIII-*ccdB*-vector. One additional premise of the design was that the screening-process for successful five-fragment assemblies should be reliable and with minimal effort. We designed a vector hat confers ampicillin, tetracycline and kanamycin resistance (Fig. 8b), if successfully assembled. The resistance to ampicillin and tetracycline depends on correct in-frame fusion of the respective PCR-fragments of the resistance genes in the assembly. Since the positive selection vector does not contribute to background in this method, we tested the contribution of PCR-template carryover by using column-purified PCR fragments without *Dpn*I digestion. We challenged the recombinogenic capacity of autoinduced PPY OTG-extract by five fragments to be joined. We first plated column-purified assemblies from five independent assembly-reactions on ampicillin-tetracycline plates and re-plated 15 colonies from each plate onto kanamycin the following day. The second plate was scored for the kanamycin-resistant colonies and the ratio of triple-resistant colonies was estimated to be 77.4% corresponding to roughly seven thousand colonies per µg transformed DNA for five assembled fragments (Fig. 8c). Finally the correspondence of the triple-resistant phenotype to the presence of pZeBRα5-plasmid was confirmed by restriction enzyme analysis with *Rsa*I of isolated plasmids (Fig. 8d). The experiment clearly demonstrates the high fidelity and recombination capacity of the SLiCE/ZeBRα-method even when PCR-carryover contributes to background. The high number of recombinants suggests that even more DNA-fragments could be joined.

## Discussion

Our main objective was to create a cloning/assembly-method that could be conveniently performed in any molecular biology lab with comparable success rates to commercial cloning and assembly kits. We have demonstrated that combining the two state-of-the-art methods, namely a “home-brewed” assembly method and the use of a zero-background (positive selection) vector synergistically improve the cloning efficiencies, provide additional flexibility while at the same time, reducing time and costs. The method that we named, ZeBRα (Zero-background Redα-mediated assembly), overcomes challenges in routine and high-throughput cloning. Currently, the main weakness of most ligation with “positive selection markers”-methods, using *ccdB*, *sacB*, (IRDL^34^: ZeBaTA^39,40^:) is the low efficiency of ligase based cloning, that does not allow seamless, multi-fragment assemblies. Recently developed methods like RapGene^41^ (positive selection marker*: Gata-1-GST*) account for this shortcoming and were demonstrated to be capable of assembling several small fragments between 57–80 bp into genes of about 800 bp with high accuracy. This is an interesting aspect of the method since commercial Gibson-assemblies are not capable of assembling DNA-fragments smaller than 150 bp (NEB, NEBuilder HiFi DNA Assembly manual). Unfortunately RapGene performance has not been demonstrated with larger fragments^41^.

SLiCE^5^ was our method of choice for the assembly-method due to its high recombinogenic capacity and the possibility to prepare unlimited amounts with minimum reagent costs. The only drawback of SLiCE is that the preparation of the PPY-extract requires significant hands-on time and expertise to ensure superior recombination capacity. Therefore, we first systematically investigated how the preparation of the recombinogenic PPY-extract can be simplified. We quantified the effect of autoinduction of Redα-exonuclease, the detergents used and the presence of detergents in the final assembly, on the number of recombinant colonies.

We were able to replicate the preparation of highly recombinogenic extracts from manually arabinose induced PPY-cells, but decided to test arabinose medium as a more convenient and reproducible alternative. The comparison of PPY-extract from Redα-exonuclease induced vs. un-induced *E. coli* clearly indicated that autoinduction increased the recombinogenic capacity under all tested extraction conditions.

Most tested non-ionic detergents were capable of producing a PPY-extract with decent recombination capacity, but OTG outperformed the other tested detergents, and was cost-effective and the extracts showed the best fidelity. The structurally very similar OG in contrast, resulted in extracts with the lowest recombinogenic capacity measured. OG was described to be particularly efficient in solubilizing the membrane bound protease OmpP^42^ a F-plasmid born homologue of OmpT. NEB 5-alpha and PPY strains are F^−^ but OmpT positive and taking into account the similarity of the proteases, it is reasonable to assume that some OmpT released by OG might account for the low recombination activity of the derived extracts.

The overall number of recombinants considerably increased if the assembly reaction was subjected to column-purification before transformation. We attribute this to the removal of the detergent, which has the potential to reduce the viability of the transformed cells.

We used commercial T4-ligase buffer (NEB) supplemented with NAD^+^ for the SLiCE/ZeBRα-assemblies (see Methods). The stimulatory effect of NAD^+^ on recombination was described for DH10B-extracts not expressing the λ-Phage recombination proteins^12^. This makes endogenous *E. coli*-ligase a candidate to be involved in the recombination events in SLiCE.

This assumption stems from comparing the proposed mechanism of SLiCE^5^ to the Gibson-assembly^1,2,43^. Both methods generate complementary overhangs in the reaction mixture by a 5′-3′ exonuclease or 3′-5′ exonuclease respectively. In case of the Gibson-assembly the gaps of annealed overhangs are filled-in by a polymerase and finally sealed by ligase. In contrast, for SliCE there is no fill-in step proposed but the concerted action of ssDNA binding Bet and the exonuclease-inhibitor Gam temporarily stabilize the annealed fragments.

To our knowledge, neither endogenous *E. coli* exonucleases nor DNA-polymerases depend upon NAD^+^ (enzyme names are followed by their UniProt accession number; ExoI: P04995, ExoIII: P09030, ExoT: P30014, ExoV: P08394, ExoVII: P04994, ExoVIII: G0FEI3, PolA: P00582, PolB: P21189, Pol IV: A7ZHZ2, PolV: P04152). Therefore we infer from the stimulatory activity of NAD^+^ that *E. coli* ligase could contribute to the recombination success by covalently sealing the nicks of the assembled DNA. Therefore the assembled DNA might be more resistant to *E. coli* exonucleases after transformation. Two recent studies^6,31^ demonstrated efficient assemblies using Phusion-Polymerase and T5-exonuclease without Taq DNA-ligase. One possible reason for this could be that the Taq DNA-ligase remaining bound to the assembled product might counter the benefit of sealing the nicks. The protein-DNA complex has an increased molecular weight thereby reducing transformation success. Since our method uses a final purification step removing proteins prior to the transformation we would only see the benefit of the ligase activity. Additional purification of ligations was shown to increase the number of transformants by the removal of T4-ligase^44–46^. The observed increase in recombinant colonies from column purified SLiCE/ZeBRα is due to the removal of the detergents and potentially the *E. coli*-ligase bound to the assembled plasmids.

We have demonstrated recombination efficiencies for both, two and three-fragment assemblies between 3–6 × 10^4^ successful recombinants/µg transformed, linear fragments with SLiCE-extracts, derived from two different *E. coli* strains (PPY, NEB 5-alpha). To our surprise the SLiCE-extract with OTG and other tested detergents derived from NEB 5-alpha worked equivalently well as the respective extract from the recombinase-expressing PPY-strain. The high efficiency of the NEB 5-alpha derived SLiCE was unexpected since we have previously shown (Fig. 3a) that PPY derived extracts from recombinase-induced cells outperformed their un-induced PPY-counterparts.

This observation might aid the acceptance of our method since DH5α-derivatives, as NEB 5-alpha, are some of the most common laboratory strains. The preparation of the NEB 5-alpha extract is significantly less laborious than a regular plasmid Midi or preparation of the autoinducing medium.

Even the recombinogenic agents in the NEB 5-alpha- extract are elusive, the high efficiency, compared to iVEC/“transformation-cloning” is apparently the result of the higher probability of two or more DNA-fragments to meet and being assembled *in vitro* than being transformed and consecutively assembled *in vivo*. The probability of co-transformation of multiple fragments into a single cell decreases, with increasing number of fragments. This inherently limits the maximal number of fragments that can be assembled *in vivo*. Additionally, we assume that the DNA assembled *in vitro* would be protected against exonucleases of the host cell, like RecBC after transformation^47^. One noteworthy observation was that iVEC/“transformation-cloning” consistently yielded error prone assemblies. The transformation of linear DNA might trigger RecA-independent SOS-response in *E. coli* as it is interpreted as DNA damage leading to error-prone repair^48,49^. Several aspects of SOS-response require transcription and translation thereby the bacterial extracts used in SliCE/ZeBRα would not be affected, explaining the higher fidelity of the method

The recombinogenic capacity of different *E. coli* cloning strains was assessed by the authors of the original SLiCE publication^5^. They found that only cell lysates from K-strains like DH10B, and particularly JM109 were capable of exerting significant recombinogenic activity *in vitro*. The reason behind the choice of the particular K- and B-strains examined was probably that the K-strains lack the major *E. coli* endonuclease (EndA) and the RecA-protein while the B-derivatives are deficient in the Lon and OmpT proteases^5^. The authors further used B-strain derivatives that are additionally either *recA*, (BLR, Novagen) or *endA* (ER2566, NEB) without any noteworthy success. This suggests that the recombinogenic capacity is either an inherent trait of K-strains or requires the absence of RecA- and EndA- activity.

Ken Motohashi’s lab investigated other *E. coli* strains and confirmed the superior recombinogenic capacity of JM109 extracts^35,50^ (*JM109: F*’ *traD36 proA*^+^*B*^+^ *lac*^*Iq*^*, Δ (lacZ)M15/Δ (lac-proAB) glnV44 e14*^*−*^ *gyrA96 recA1 relA1 endA1 thi hsdR17 lon::IS186-putative!*). A personal communication to OpenWetware (https://openwetware.org) implies that in contrast to the commonly assumed genotype, JM109 might be deficient in the Lon protease due to an IS186 transposon insertion (Juergen Mairhofer; University of Natural Resources and Applied Life Sciences, Vienna; Vienna Institute of BioTechnology (VIBT)). If this transposon-insertion is present in all JM109 stocks it might be a reasonable explanation of the high recombinogenic activity of the derived extract by sparing relevant proteins from degradation.

The authors also included a W-strain (*Mach1 T1R: F*^*−*^ *φ80lacZΔM15 ΔlacX74 hsdR(rK*^*−*^*, mK*^+^) *ΔrecA1398 endA1 tonA*) derived extract that exerted significant recombinogenic capacity.

The smallest common denominator of the examined strains producing significantly recombinogenic extracts therefore seems to be the absence of EndA, and certain aspects RecA-dependent processes. An interesting observation we made is that *E. coli* K-12 and W-strains are recE^+^ and recT^+^ while B-strains are lacking the RecE-exonuclease. (BL21D GenBank: CP010816.1 (https://goo.gl/bDf29z), NEB 5-alpha GenBank: CP017100.1 (https://goo.gl/jmigac), W-strain NCBI Reference Sequence: NC_017635.1 (https://goo.gl/5iM7hJ)). If this could account for the described differences between the recobinogenic capacity between the different strains remains to be tested.

The transformation cloning also referred to as iVEC (*in Vivo E. coli* Cloning) has been described more than 25 years ago^29,30^ and was used sporadically ever since. The underlying enzymes were only very recently identified as ExoIII (XthA) and PolA^51^. The authors elegantly narrowed down the key enzymes and also demonstrated that RecA and RecET are not essential for iVEC. This carefully designed study further demonstrated that the benefit of *recA* is to reduce plasmid multimerization, rather than forcing the usage of an alternative recombination pathway. Based upon these results the authors constructed a *recA endA hsdR* derivative of MG1655 for iVEC. This is in accordance with our experience with NEB5-alpha strain that is a *recA endA hsdR* K12 strain related to MG1655 and performant in promoting iVEC and SLiCE assemblies. It is likely that the sets of enzymes promoting iVEC and SLiCE overlap; therefore, the mechanistic insight into iVEC could also be an important step in understanding the mechanisms underlying SLiCE. As described in the present paper the DH10B derived PPY strain showed virtually no assembling-capacity if used for iVEC, despite being wildtype regarding the highly conserved *exoIII/xthA*. This also holds true for all sequenced B- and W-strains thereby making ExoIII/XthA potentially necessary but not sufficient to promote SLiCE (ExoIII: NEB5-alpha https://goo.gl/qJQ29z, W-strain https://goo.gl/J5nZg6, BL21(DE3) https://goo.gl/iDYaTy). It will be very interesting to test the performance of a detergent extract of the *xthA-*mutant and the novel iVEC-strain in a SLiCE setup.

In SLiCE, the extracted bacterial cellular content in the assembly reaction mixture is likely to create molecular-crowding, thereby aiding the assembly. For ligase based cloning, reagents like PEG 6000–8000 (polyethylene glycol)^52^ and hexamine cobalt chloride^53^ are used to increase macromolecular crowding for increased ligation efficiency. Addition of PEG 6000 to our reaction-buffer did not further enhance assembly efficiency (data not shown).

A recent study created a porcine *ccdB*-expressing vector and used it with a SLiCE extract^54^ prepared according to the original protocol^5^ successfully.

In contrast, our approach optimized the SLiCE preparation protocol to make it more convenient and robust with fewer hands-on steps. We have systematically examined the most critical steps for successful multi-fragment assembly and avoided expensive reagents as the CelLyticB^TM^. Our small riboprobe vector thereby serves a dual purpose. Firstly it is intended to replace TOPO®/TA-cloning vectors for sequencing and *in vitro* transcription. Secondly, the toxic placeholder *ccdB*-cassette can be transplanted into any other vector qualifying it for zero-background-cloning.

ZeBRα-assemblies are of particular interest where huge vectors are used, as in the case of transgenes for *Drosophila*, zebrafish, mice, plants and cell culture. It is common practice to sequentially assemble all fragments-of-interest in a small “cloning-vector” and subsequently sub-clone the assembled product “en bloc” into the “expression-vector”. This laborious approach led to the widespread use of commercial cloning systems like Gateway® where multiple fragments can be assembled in fewer steps and higher efficiency compared to ligase based cloning. The convenience and efficiency of these products lead to their widespread use but that comes at a high price (250–350€/20-reactions, for multisite-Gateway® 2400€ as of 2018). SLiCE mediated multi-fragments assemblies are scar less, require fewer steps and are more flexible compared to multisite-Gateway® with the commercial recombinases supplied.

One of the greatest advantages of ZeBRα as a strategy is that SLiCE-extracts are compatible with the existing non-commercial collections of *ccdB*-cassette bearing vectors. This expands the range of ZeBRα-applications to virtually all model-organisms on the spot (for a comprehensive list of non-commercial collections of *ccdB*-cassette vectors see: https://blog.addgene.org/plasmids-101-gateway-cloning). Additionally, integrating the *ccdB*-cassette into any vector of choice can create customized vectors. Since the ZeBRα-vector was derived from Heaven’s Gate-cloning, it is fully compatible with Golden-Gate^7,8^ and all related methodologies, exploiting the unique features of Type IIS-restriction enzymes.

Linearized ZeBRα-vectors as well as the recombinogenic extracts can be stored and used off-the-shelf, simplifying the “cloning pipeline” and allowing a high level of parallelization.

The high efficiency and low cost of ZeBRα helps in resource allocation by significantly saving hands-on time as well as budget. We hope that employing the powerful new methods of synthetic biology through ZeBRα will provide researchers an additional degree of freedom and flexibility for high-throughput and routine cloning.

## Methods

### Materials

Restriction enzymes and T4 DNA ligase-buffer were from New England Biolabs (NEB). All detergents, antibiotics and reagents were purchased from Carl Roth, Karlsruhe, except the branched chain amino acids were purchased as blend from Pumpeffect B.V. Maastricht.

### PPY-lysate activity measurement

In order to assess the effect of induction and purification on the assembling capacity of the PPY-lysate we slightly modified the method described in^11^. In brief, the assembled plasmid drives the constitutive expression of a blue chromoprotein and allows colony formation on kanamycin-plates. The number of blue cfu/µg transformed total linear DNA is used as a measure of the assembling capacity of the respective PPY-extract.

### Generation of Test Fragments for PPY-Extract testing by PCR

The linear PCR-products used test the assembling capacity of the PPY-lysates were generated by PCR from the vectors listed in Tables S7 and S8. The amplicons for the three-fragment-assembly consisted of the promoterless blue chromoprotein (insert), a fragment consisting of the lac-promoter and a fragment consisting of the pUC-origin of replication and the gene encoding aminoglycoside phosphotransferase driven by its own promoter, conferring kanamycin resistance (vector). In the four-fragment-assembly the gene encoding aminoglycoside phosphotransferase (KanR) and the pUC-origin were separate Fragments. All PCR-Fragments were generated with Q5 polymerase (NEB) and 1 ng template DNA.

### Preparation of Chemically Competent NEB 5-alpha *E. coli*

Chemically competent NEB 5-alpha *E. coli* cells were prepared according to the method described by Inoue^21^. Cells were aliquoted á 150 µl, transformation efficiency was estimated to be 2.3 × 10^6^ cfu/µg DNA. To ensure constant transformation efficiency throughout the study, a single batch of cells was used for the experiments except those shown in Fig. 5. The NEB 5-alpha *E. coli* batch used in Fig. 5 had a competency of 2.5 × 10^8^ cfu/µg, tested by transformation of pT7-GFP-antisense. This respective batch of NEB 5-alpha was prepared by the Inoue-method where the SOB-medium used to culture the cells and the outgrowth-medium contained 10 g glycine/l.

### Autoinduction of Redα in PPY cells with arabinose and preparation of cell lysates

A single PPY-colony^8^ (F- *end*A1 *rec*A1 *gal*E15 *gal*K16 *nup*G *rps*LΔ*lac*X74 Φ*80lac*ZΔM15 *ara*D139Δ(*ara,leu*)7697 *mcr*A Δ(*mrr-hsd*RMS*-mcr*BC) *cyn*X*::* [*ara*C pBAD*- red*α EM7*- red*βTn5*-gam*]^***−***^*)* was inoculated in 10 ml PAG-Medium (Table S1) over night at 37 °C with shaking at 250–300 rpm. Baffled Erlenmeyer-flasks containing 50 ml Studier’s autoinduction medium^20^ supplemented with arabinose or lactose (see Tables S3 and S4) were inoculated with 150 µl of the PPY overnight culture. All cultures were grown at 37 °C with shaking at 350 rpm over night until the OD did not further increase (OD600 ≈ 15). Six independent replicates of the autoinduction culture and four replicates for the lactose mock-induction were treated as described and pooled prior to harvesting. Cells were harvested by centrifugation (4.600 rpm, 4 °C, 15 min). The cell pellet was re-suspended in 50 ml PBS aliquoted to contain the same amount of cells and pelleted (4.600 rpm, 4 °C, 15 min). For preparation of the PPY extracts the aliquoted pellets were re-suspended in the respective detergent solution to be tested (300 µl detergent solution per 0.23 g wet-pellet weight). Each detergent was dissolved in Tris-HCl buffer, pH 7,5 at a concentration of 1% (w/v), which corresponded to a concentration above the CMC (Critical Micelle Concentration). The cell pellets were re-suspended by pipetting and the suspension was incubated (low binding tubes #72.706.600, Sarstedt, Nümbrecht) for 10 min at room temperature for lysis. Cell lysates were centrifuged (10.000 g, room temperature, 2 min) subsequently. The resulting supernatants, representing the final cell extract, were aliquoted á 300 µl and mixed with 300 µl of 100% glycerol. The cell extracts were stored at −80 °C.

### SLiCE-extract preparation with multiple detergents from JM109 and NEB 5-alpha

JM109 cells were plated onto LB (all media contained 30 µg/ml nalidixic acid). A single colony was used to inoculate 4 ml 2YT and incubated over night at 37 °C/250 rpm. 1 ml of the overnight-culture was used to inoculate 3 × 50 ml 2YT in 250 ml baffled Erlenmeyer flasks and cultivated until the OD_600_ reached approximately 2,3. Three cultures were mixed and cells were harvested by centrifugation at 3400 g (4000 rpm) for 15 min at 4 °C. Cell pellets were re-suspended in 12 ml sterile 1x PBS and aliquoted a 1.5 ml into low protein binding Eppendorf tubes.

The cells were pelleted at 11 000 rpm for 1 min. 0,07 g cell-pellet was re-suspended in 200 µl of the respective detergent solution. 1% (w/v) detergent in 50 mM Tris-HCl pH 7.5 corresponding to a concentration of the CMC or above was used to gently re-suspend the pellet with a pipette tip until the suspension was homogenous. The suspension was incubated for 10 minutes at RT. Debris was removed by centrifugation for 2 minutes at 13500 rpm and the supernatant was aliquoted into low-protein binding tubes. None of the detergents released chromosomal DNA, inferred from the low viscosity of the solution, indicating an incomplete lysis.

The supernatant was mixed with an equal volume of 100% glycerol. Finally the extracts were frozen by placing them into a pre-cooled brass-rack at −80 °C.

### Assembly of DNA fragments by SLiCE

In case of three-way assembly the assembly reaction was set up with 100 ng Vector and 3-fold molar excess of inserts (in case of four-way assembly, the ratios were 1:1:1:1), 1 µl 10x T4 DNA ligase buffer (NEB), 1 µl NAD^+^ (260 µM), 1 µl PPY-extract and ddH_2_O to a total volume of 10 µl. The reaction was incubated for 30 minutes at room temperature. Prior to transformation, the assembly reaction was purified using the Oligo Clean & Concentrator^TM^ Kit (Zymo Research, Irvine).

### Transformation of NEB 5-alpha with assembled Plasmids and measuring the recombination capacity of the PPY extracts

Frozen chemically competent NEB 5-alpha (DH5α–derivative, NEB) cells (2.3 × 10^6^ cfu/µg) were thawed on ice. A volume corresponding to 200 ng total DNA from the purified assembly was added to 100 μl bacterial suspension and incubated on ice for 30 minutes. Cells were heat shocked subsequently at 37 °C for 30 seconds and followed by incubation on ice for 2 minutes. 900 μl SOC-medium without antibiotic was added and the suspension was incubated at 37 °C for one hour under constant agitation (280 rpm). After the recovery, 80–100 µl of the cell suspension was plated on LB- agar plates (with antibiotics) and incubated at 37 °C over night. Colonies of successful recombinants were identified by their intense blue color or GFP fluorescence and counted semi-automatically with the particle analyzer in Fiji^39^.

## Supplementary information

Supplements

## Data Availability

The data generated during and/or analyzed during the current study are available from the corresponding author on reasonable request.
