# Supplementary material for: ZeBRα a universal, multi-fragment DNA-assembly-system with minimal hands-on time requirement
Source: Sci Rep. 2019 Feb 27;9:2980. doi: 10.1038/s41598-019-39768-0 (PMC6393441; doi:10.1038/s41598-019-39768-0)
Supplement: Supplementary file 1 — Supplements [file 41598_2019_39768_MOESM1_ESM.docx]

**ZeBRα a universal, multi-fragment DNA-assembly-system with minimal hands-on time requirement**

David Richter^1*^, Katharina Bayer^1^, Thomas Toesko^1^& Stefan Schuster^1^

^1^Department of Animal Physiology, University of Bayreuth, 95440 Bayreuth, Germany.

**Supplementary Information**

**
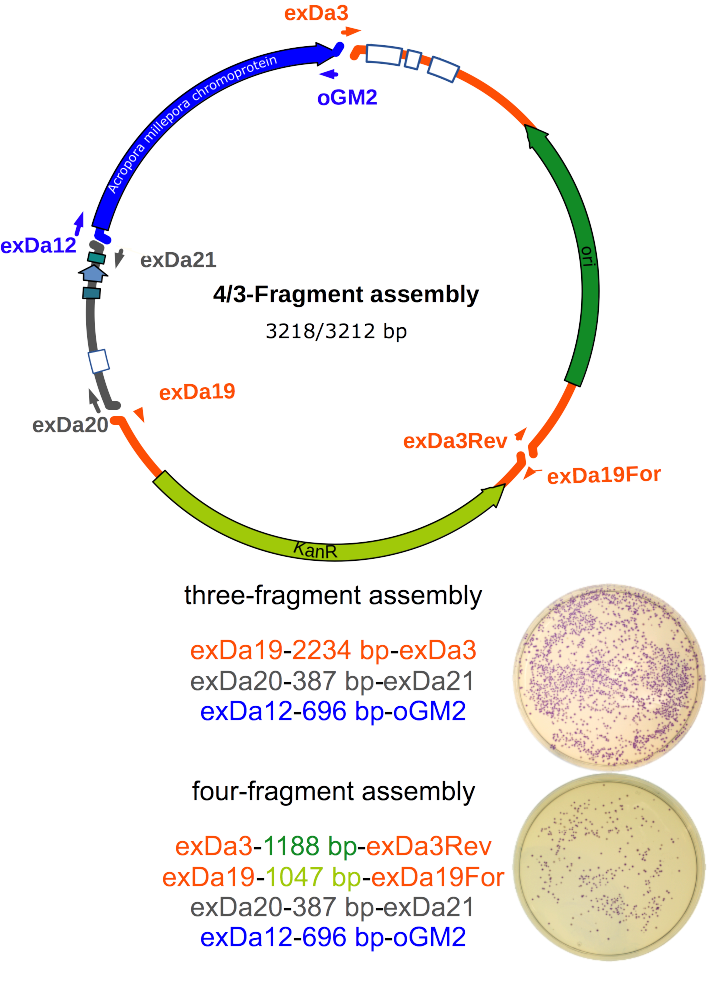
**

*Figure S1.* **Fragments used in three- and four-fragment assemblies.** Plasmid map of three- and four-way assemblies and the used PCR-fragments. A blue chromoprotein was used as readout for the potency of the PPY extracts. The three-fragment- assembly consists of three PCR-fragments with about 15 bp overlapping ends. The fragments correspond to a kanamycin resistant vector backbone, a promoter and the ORF coding for the blue chromoprotein. In the case of a four-fragment-assembly, the plasmid vector was recombined from two separate PCR-fragments. The number of blue colonies that appear after transformation is proportional to the recombination capacity of the cell extract (lower left corner)


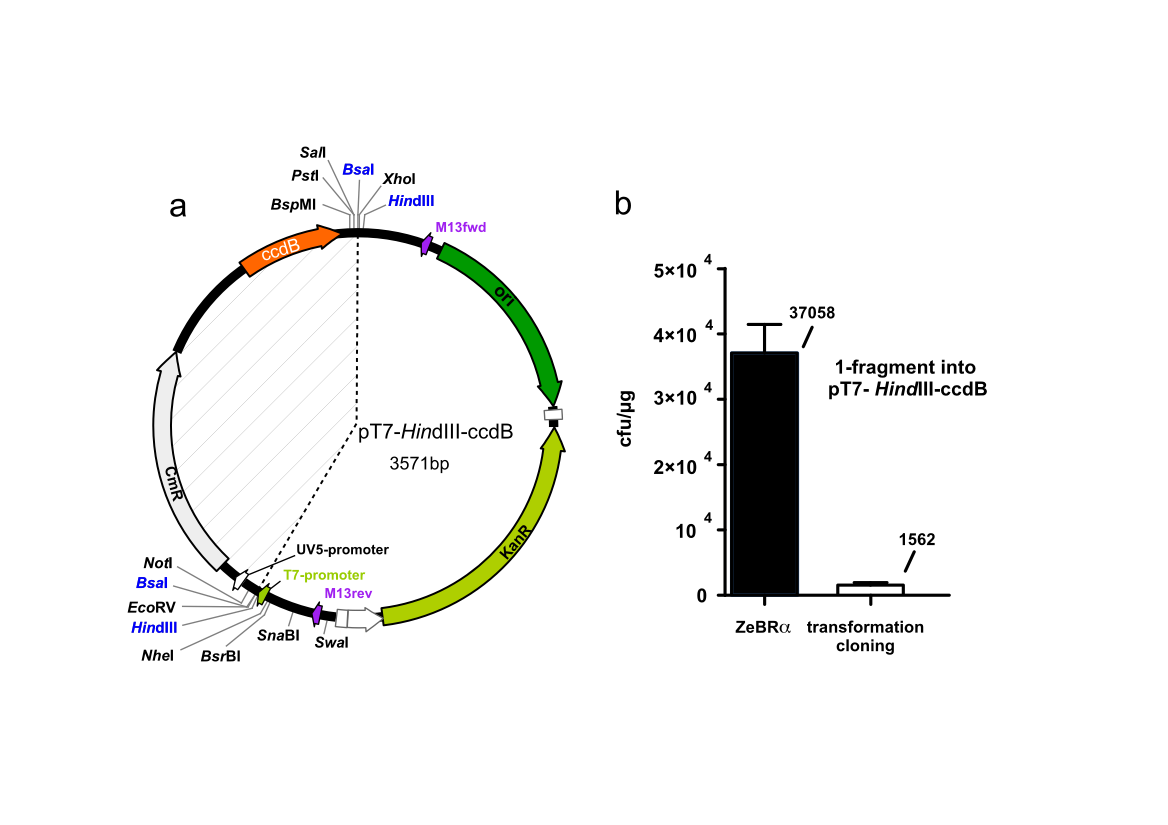


*Figure S2.* **Plasmid map and performance of pT7-*Hin*dIII-*ccdB* in ZeBR**α **cloning. a*,*** Two *Hin*dIII and two *Bsa*I sites flank the toxic-placeholder- *ccdB,* allowing convenient linearization and removal of *ccdB*. Unique sites are available on either side of *ccdB* (bold). Chloramphenicol acetyl-transferase coding gene (*CmR*), is part of the placeholder cassette and prevents *ccdB*-loss during plasmid propagation. The dotted lines encompass the region removed during cloning. **b,** ZeBRα: PPY-extract mediated assembly of a 700 bp PCR-fragment with 15 bp identical ends shared with the vector into pT7-*Hin*dIII-*ccdB* is about 23 times more efficient than “transformation-cloning”

*Figure S3.* **Cell extracts derived from *E. coli* K12 strains have high detergent dependent recombinogenic capacity without ectopic expression of phage proteins.** **a,** NEB 5-alpha extracts of four detergents and the commercial CelLytic were tested for their ability to join three PCR-fragments resulting in a plasmid conferring blue colony phenotype or white colonies indicating defective assemblies as described for PPY cells.

Extracts derived from NEB 5-alpha produced more than 10^4^ cfu/µg with about 1-4% defective colonies. CHAPS and SB-12 were more efficient than the other detergents but also produced slightly more defective colonies. All extracts were prepared from a blend of three independently fermented cultures **b,** JM109 extracts of four detergents and the commercial CelLytic were tested for their ability to join three PCR-fragments resulting in a plasmid conferring blue colony phenotype or white colonies indicating defective assemblies as described for PPY cells. Extracts derived from JM109 produced at least 10^4^ cfu/µg. CHAPS being the least effective and CelLytic and OTG being the most recombinogenic producing almost 3x10^4^ cfu/µg. Generally JM109 derived extracts produced more defective colonies than NEB 5-alpha extracts. All extracts were prepared from a blend of three independently fermented cultures. All assemblies were column-purified before transformation into NEB 5-alpha.


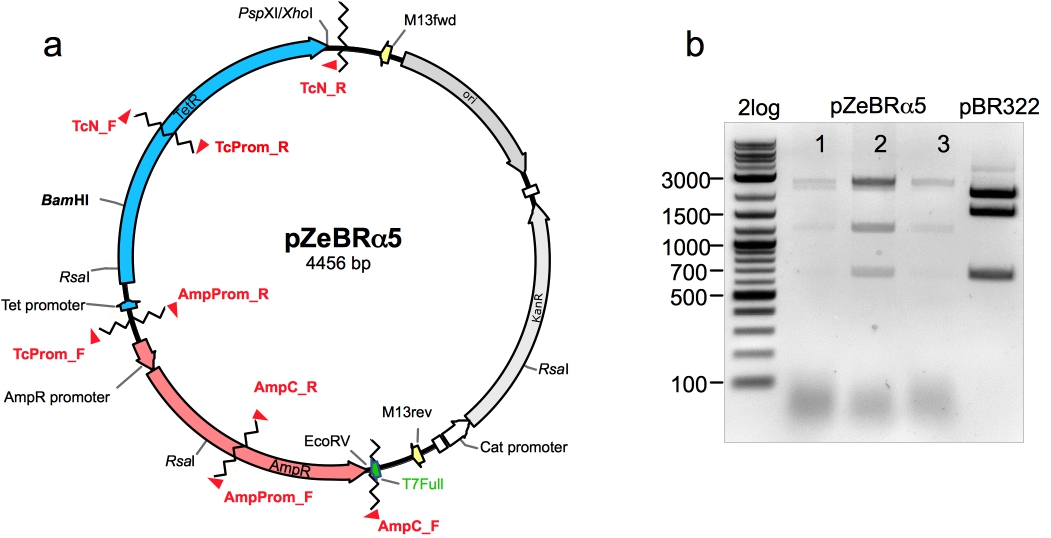


*Figure S4.* **Five-fragment ZeBRα-assembly for estimating the fidelity of the reaction results in the triple-antibiotic resistant pZeBR**α**. a*,*** Plasmid map of the pZeBRα. Selected restriction enzyme recognition sites are shown in black. PCR-primers used for amplification of assembled fragments and their respective binding sites (arrowheads) are shown in red. The blue and red arrows indicate the *bla* and *tetA* genes conferring resistance to ampicillin and tetracycline respectively. The criss-cross line indicates the fusion sites of the individual fragments. The plasmid backbone is derived from pT7-*Hin*dIII-*ccdB Hin*dIII **b,** characteristic *Rsa*I digestion pattern of pZeBRα and pBR322, latter served as template for generating the cloned fragments.


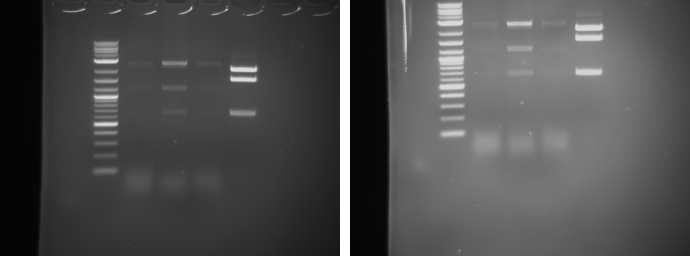


*Figure S5.* **Five-fragment ZeBRα-assembly confirmation of the pZeBR**α**- vector identity conferring triple-antibiotic resistant by restriction enzyme digestion. a*,*** the gel-images are unedited raw images displaying the result of the restriction enzyme digestion seen in Figure S4.

*Table S1/S2.* **CCMB80 chemically competent PPY cells are not suitable for “transformation-cloning”.**

_Transformation-cloning PPY_ 70 ng/µl linear Vector

| cfu/µg total | **cfu/µg GFP^+^** |
| --- | --- |
| 960 | **0** |
| 1000 | **0** |
| 360 | **40** |
| 1280 | **40** |

pT7-Hind-ccdB (***120** ng/µl) *Bsa*I-FastAP-column-purified

lacP+RBS 50 ng/µl- 280 bp

GFP-ORF 50 ng/µl -760 bp

***Corresponding to 70 ng/µl linear Vector**

_Transformation-cloning PPY_ 29.5 ng/µl linear Vector

| cfu/µg total | **cfu/µg GFP^+^** |
| --- | --- |
| 1680 | **120** |
| 1480 | **80** |
| 0 | **0** |

pT7-Hind-ccdB (***50** ng/µl) *Bsa*I-FastAP-column-purified

lacP+RBS 50 ng/µl- 280 bp

GFP-ORF 50 ng/µl -760 bp

***Corresponding to 29.5 ng/µl linear Vector**

*Table S3.* **Modified non-inducing PAG medium for PPY.**

| Amount | Component |
| --- | --- |
| 9.07 ml | ddH_2_O |
| 20 µl | 1M MgSO_4_ |
| 1 µl | 1M FeCl_3_ in 1.2M HCl |
| 125 µl | 40%(w/v) glucose |
| 500 µl | 20xP |
| 125 mg | Leucine |
| 62.5 mg | Isoleucine |
| 62.5 mg | Valine |
| 10 µl | Streptomycin (50 mg/ml) |

*Table S4.* **Complete autoinduction medium used for induction of Redα.**

| Amount | Component |
| --- | --- |
| 185.6 ml | ZY-Medium |
| 0.4 ml | 1M MgSO_4_ |
| 0.02 ml | 1M FeCl_3_ in 1.2M HCl |
| 4 ml | 50x 5052-Arabinose |
| 10 ml | 20x P |
| 0.05 ml | Streptomycin (50 mg/ml) |

*Table S5.* **Composition of inducing 50x 5052-Arabinose medium.**

| Amount | Component |
| --- | --- |
| 25 g | Glycerol |
| 73 ml | H_2_O |
| 2.5 g | Glucose |
| 10 g | L-(+)-Arabinose |

*Table S6.* **Composition of non-inducing 50x 5052-Lactose medium.**

| Amount | Component |
| --- | --- |
| 25 g | Glycerol |
| 73 ml | H_2_O |
| 2.5 g | Glucose |
| 10 g | L-(+)-Lactose |

*Table S7.* **Composition of 20xP.**

| Amount | Component |
| --- | --- |
| 90 ml | ddH_2_O |
| 14.2 g | Na_2_HPO_4_ |
| 13.6 g | KH_2_PO_4_ |
| 6.6 g | (NH_4_)_2_SO_4_ |

pH of 50-fold dilution should be ~6.7

*Table S8.* **ZY-Medium.**

| Amount | Component |
| --- | --- |
| 10 g | tryptone |
| 5 g | Yeast-extract |

Ad 1 liter H_2_O

*Table S9.* **PCR-Conditions for preparing the DNA-fragments used for testing the PPY extract.**

| 3-fragment  assembly | Fragment | Template  Primer | PCR conditions |
| --- | --- | --- | --- |
|  | insert | pSB1C3-K592009  oGM12  oGM2 | 98°C for 30 s; 98°C for 10 s; 70°C for 20 s; 72°C for 30 s (repeated for 28 cycles); 72°C for 2:00 |
|  | promoter | pSB1C3-J04450  exDa20  exDa21 | 98°C for 30 s; 98°C for 10 s; 60°C for 20 s; 72°C for 15 s (repeated for 28 cycles); 72°C for 2:00 |
|  | ori | pSB1K3-J04450  exDa3  exDa19 | 98°C for 30 s; 98°C for 10 s; 62°C for 20 s; 72°C for 1:40 (repeated for 25 cycles); 72°C for 2:00 |

| 4-fragment assembly | insert | pSB1C3-K592009  oGM12  oGM2 | 98°C for 30 s; 98°C for 10 s; 70°C for 20 s; 72°C for 30 s (repeated for 28 cycles); 72°C for 2:00 |
| --- | --- | --- | --- |
|  | promoter | pSB1C3-J04450  exDa20  exDa21 | 98°C for 30 s; 98°C for 10 s; 60°C for 20 s; 72°C for 15 s (repeated for 28 cycles); 72°C for 2:00 |
|  | KanR | pSB1K3-J04450  exDa19.For  exDa19 | 98°C for 30 s; 98°C for 10 s; 68°C for 15 s; 72°C for 30 s (repeated for 25 cycles); 72°C for 2:00 |
|  | ori | pSB1K3-J04450  exDa3  exDa3.Rev | 98°C for 30 s; 98°C for 10 s; 70°C for 15 s; 72°C for 30 s (repeated for 25 cycles); 72°C for 2:00 |

*Table S10.* **PCR-Conditions for preparing the DNA-fragments for ZeBRα.**

| ZeBRα-assembly | Fragment | Template  Primer | PCR conditions |
| --- | --- | --- | --- |
|  | GFP 760 bp | UAS-PSD95-GFP  ZebraGFP_Fw  ZebraGFP_Rw | 98°C for 30 s; 98°C for 10 s; 64°C for 20 s; 72°C for 30 s (repeated for 32 cycles); 72°C for 2:00 |
|  | promoter 280 bp | pSB1K3-J04450  ZebraProm_Fw  ZebraProm_Rw | 98°C for 30 s; 98°C for 10 s; 62°C for 20 s; 72°C for 20 s (repeated for 32 cycles); 72°C for 2:00 |
|  | vector | **pT7-*Hin*dIII-ccdB** | *Bsa*I-digested, FastAP-dephosphorylated-column-purified (120 ng/µl) |

*Table S11.* **PCR-Conditions for preparing the DNA-fragments for ZeBRα5.**

| ZeBRα-assembly | Fragment | Template  Primer | PCR conditions |
| --- | --- | --- | --- |
|  | Tc1 690 bp | pBR322  TcN_F  TcN_R | 98°C for 1min; 98°C for 10 s; 66°C for 25 s; 72°C for 25 s (repeated for 30 cycles); 72°C for 2:00 |
|  | Tc2 647bp | ****Tc 671 bp**  TcProm_F  TcProm_R | 98°C for 1min; 98°C for 10 s; 67°C for 25 s; 72°C for 25 s (repeated for 30 cycles); 72°C for 2:00 |
|  | Ap1 641 bp | pBR322  AmpProm_F  AmpProm_R | 98°C for 1min; 98°C for 10 s; 66°C for 25 s; 72°C for 25 s (repeated for 30 cycles); 72°C for 2:00 25 |
|  | Ap2 480 bp | pBR322  AmpC_F  AmpC_R | 98°C for 1min; 98°C for 10 s; 66°C for 25 s; 72°C for 25 s (repeated for 30 cycles); 72°C for 2:00 |
|  | vector | **pT7-*Hin*dIII-ccdB** | *Bsa*I-digested, FastAP-dephosphorylated-column-purified (120 ng/µl) |

| ZeBRα-assembly | Fragment | Template  Primer | PCR conditions |
| --- | --- | --- | --- |
|  | ****Tc 671bp** | pBR322  TcProm_F  TcProm_R1 | 98°C for 1min; 98°C for 10 s; 67°C for 25 s; 72°C for 25 s (repeated for 30 cycles); 72°C for 2:00 |

*Table S12.* **Primer Sequences used, naming as in^22^.**

| Primer | Sequence |
| --- | --- |
| oGM2 | **ATTTGATGCCTGG**TTATTAGGCGACCACAGGTTTGCGTGC |
| exDa3/oGM3 | **GGTCGCCTAATAA**CCAGGCATCAAATAAAACGAAAGGCT |
| *exDa3 Rev | **GACTCGAG**CTCGAGCTGTCAGACCAAGTTTACT |
| oGM12 | **AGGAGAAATACT**AGATGAGTGTGATCGCTAAACAAATGACCTACAAGG |
| exDa20 | **TGAGTTGAAGGATCAGCT**TCTAAGAAACCATTATTATCATGACATTAACC |
| exDa21 | **AGCGATCACACTCATCT**AGTATTTCTCCTCTTTCTCTAGTATGTG |
| exDa19 | **TGGTTTCTTAGA**GCTGATCCTTCAACTCAGCA |
| *exDa19For | **GCTCG**AGCTCGAGTCCCGTCAAG |
| *ZebraGFP_Fw | **GAAATACTAG**CCATGGTGAGCAAGGG |
| *ZebraGFP_Rw | **GGAGAAGCTTGCGATATC**CGCGGCCGCTTT |
| *ZebraProm_Fw | **CCAAGCTTTTTAAACTCGAG**TGGAATTCGCGGCCG |
| *ZebraProm_Rw | **CTCACCATGG**CTAGTATTTCTCCTCTTTCTCTAGTATG |
| *AmpC_F | **GGAGAagcttgcGATATC**GGTCTGACAGTTACCAATGCTTAATC |
| *AmpC_R | **TGACAACGATCGGAGGACCG** |
| *AmpProm_F | **CCTCCGATCGTTGTCAGAAGTAAG** |
| *AmpProm_R | **CATGAGAATTCTTGAAGACGAAAGGG** |
| *TcProm_F | **CGTCTTCAAGAATTCTCATGTTTGACAG** |
| *TcProm_R | **ACTCCTGCATTAGGAAGCAGC** |
| *TcN_F | **GCTTCCTAATGCAGGAGTCGC** |
| *TcN_R2 | CGCTGCAGCCATTCAGGTCGAGGTGG |
| *TcN_R1 | CCAAGCTTTTTAAACTCGAGCGCTGCAGCCATTCAG |
|  |  |

* denotes primers designed for this study

*Table S13*. **Composition of SLiCE/ZeBRα-assembly.**

| Component | Reagents |
| --- | --- |
| *E. coli* extract | n-Octyl-β-D-thioglucopyranosid, 1%(w/v) in 50 mM Tris-HCl pH 7.5 |
| T4-ligase-buffer | #B0202S, (New England Biolabs) |
| NAD^+^ | 10 mM in H_2_O (Carl Roth) |
| H_2_O | MilliQ autoclaved |
| vector | **pT7-Hind-ccdB** (120 ng/µl) *Bsa*I-digested, FastAP-dephos.-column-purified |
| insert 1 | lacP+RBS 50 ng/µl- 280 bp column-purified |
| insert 2 | GFP-ORF 50 ng/µl -760 bp column-purified |

*Table S14.* **SLiCE/ZeBRα-assembly.**

_ZeBR_α

| Component | Amount |
| --- | --- |
| *E. coli* extract/PPY or NEB 5-alpha | 1 µl |
| T4-ligase-buffer | 1 µl |
| NAD^+^ | 1 µl |
| H_2_O | 4 µl |
| vector | 1 µl |
| insert 1 | 1 µl |
| insert 2 | 1 µl |

Σ 10 µl

incubate 30’ RT

column-purification

elute in 8 µl, transform into NEB 5-alpha

*Table S15.* **SLiCE/ZeBRα-assembly.**

_ZeBR_α5

| Component | Amount |
| --- | --- |
| *E. coli* extract/PPY or NEB 5-alpha | 1 µl |
| T4-ligase-buffer | 1 µl |
| NAD^+^ | 1 µl |
| H_2_O | 2 µl |
| vector | 1 µl |
| Fragment-1 | 1 µl |
| Fragment-2 | 1 µl |
| Fragment-3 | 1 µl |
| Fragment-4 | 1 µl |

Σ 10 µl

incubate 35’ RT

column-purification

elute in 8 µl, transform into NEB 5-alpha

Inserts were 100 ng/µl each

*Table S16.* **Transformation-cloning.**

_Transformation-cloning_

| Component | Amount |
| --- | --- |
| vector | 1 µl |
| insert 1 | 1 µl |
| insert 2 | 1 µl |

Σ 3 µl

incubate 30’ RT

transform into NEB 5-alpha

Remarks:

- Column-purification is the main source of variance, the amount of recovered DNA varied considerably.
- Loading the flow through repetitively, using the maximum volume binding buffer and reloading the eluted DNA onto the column in the final step moderately increased the recovery rates.
- Increasing the DNA content of the assembly reactions increases the number of colonies.
- SOC-Medium with glycine as outgrowth medium is beneficial.
- Plating all cells increases the colony numbers. Therefore after the incubation in the outgrowth-medium cells are gently spun down at 5000 rpm for 1’, the medium is decanted and the cells are gently re-suspended in 100 µl SOC or LB for spreading.
